# Designing for culturally responsive social robots: An application of a participatory framework

**DOI:** 10.3389/frobt.2022.983408

**Published:** 2022-10-20

**Authors:** Belinda Louie, Elin A. Björling, Annie Camey Kuo, Patrícia Alves-Oliveira

**Affiliations:** ^1^ School of Education, University of Washington-Tacoma, Tacoma, WA, United States; ^2^ Human Centered Design and Engineering, University of Washington, Seattle, WA, United States; ^3^ Center to Support Excellence in Teaching, Stanford University, Palo Alto, WA, United States; ^4^ Paul Allen Center for Computer Science, University of Washington, Seattle, WA, United States

**Keywords:** social robots, english language learners, child-robot interaction, cultural responsiveness, educational robotics

## Abstract

Integrating cultural responsiveness into the educational setting is essential to the success of multilingual students. As social robots present the potential to support multilingual children, it is imperative that the design of social robot embodiments and interactions are culturally responsive. This paper summarizes the current literature on educational robots in culturally diverse settings. We argue the use of the Culturally Localized User Experience (CLUE) Framework is essential to ensure cultural responsiveness in HRI design. We present three case studies illustrating the CLUE framework as a social robot design approach. The results of these studies suggest co-design provides multicultural learners an accessible, nonverbal context through which to provide design requirements and preferences. Furthermore, we demonstrate the importance of key stakeholders (students, parents, and teachers) as essential to ensure a culturally responsive robot. Finally, we reflect on our own work with culturally and linguistically diverse learners and propose three guiding principles for successfully engaging diverse learners as valuable cultural informants to ensure the future success of educational robots.

## 1 Introduction

International migrants have increased by 62.4% during the last two decades, from 173 million to 2000 to 281 million in 2021 with over 41 million of them being children under 18 (McAuliffe et al., 2019). Although more than half of all migrants (141 million) live in Europe and Northern America, in total, they reside in 226 countries across the globe (United Nations, 2020). The issue of integration of child migrants is a major concern. Providing language support is a key element during this integration process. Facing language barriers, migrant students often perform at significantly lower language levels than their native peers in their academic performance in literacy despite their strong motivation to learn (OECD, 2021). It is tough to grow academically when some migrant students do not have adequate proficiency in the language of instruction (Barajas-Lopez, 2009; Torres-Guzman, 2017; Dewaele, 2019). With the shortage of well-trained teachers for a large, vulnerable, and high-need population in Europe and in the US (Dronkers and de Heus, 2016; Koglbauer, 2018; U.S. Department of Education, 2019; unicef, 2019), the deployment of educational robots is a viable technological solution to provide support. Many educators have voiced the necessity of teaching these learners in a culturally appropriate and responsive manner, confirming their identities, treating their cultural differences as assets not deficits, and giving them agency and control over the learning tasks to build efficacy and confidence (Powell et al., 2016; Gay, 2018; Grant and Ray, 2018; Khalifa, 2018; Ford, 2021). For robot designers, the challenge lies in how to design culturally responsive robots when designers often have a very limited understanding of the diverse cultures of language learners (Sun, 2020).

In this paper, our interdisciplinary team of applied linguists, roboticists and technology designers argue that classroom-related cultural factors must be considered in the design of educational robots and child-robot interaction. Cultural awareness and responsiveness are necessary in any support for culturally and linguistically diverse learners. There is no “one size fits all” approach. Given the incredible diversity across language learners and educational settings, it is likely that an educational robot designed specifically for one cultural group will not engage learners from very dissimilar cultural background. For social robots to be effective in classrooms, designers for robotic features/embodiment and interactions should integrate cultural responsiveness into the design process. Culturally responsive strategies and approaches have been promoted in language instruction, however, such strategies have yet to be fully integrated into the design of new technologies including educational robots (Sun, 2012; Ladson-Billings, 2014; Gay, 2018). In this paper, we discuss the rationale for a participatory approach to social robot design. We then present three case studies [two of which have been published in greater detail (Björling et al., 2021; Louie et al., 2021)] as illustrations of three distinct principles for working with culturally and linguistically diverse children.

## 2 Culturally diverse language learners

Lack of proficiency in the language of instruction is the primary reason for poor academic performance among newly arrived migrant students. It is essential for the newcomers to be capable of following lessons in the language used at school (Christensen and Stanat, 2007; Martin and Suárez-Orozco, 2018; Gándara and Contreras, 2020). Furthermore, when migrant students are not proficient in the language of instruction, they are sometimes placed into remedial classes or in special education classes (Sullivan, 2011; Gámez, 2019; National Center for Learning Disabilities, 2020). Therefore, it is fundamental that schools provide sufficient language instruction support for migrant children, and that teachers receive effective training to be able to teach the host language (Meehan et al., 2021). It is well documented that many language learners, who come from migrant families, are caught in a sociocultural struggle (Suárez-Orozco and Suárez-Orozco, 2002; Lee and Zhou, 2015; McFarland et al., 2019). They come to the United States to escape poverty and persecution and to improve the general quality of their lives (Gay, 2018). In doing so, they often suffer deep affective losses of supportive networks and familial connections. The geographic, cultural, and psycho-emotional uprootedness causes feelings of vulnerability and insecurity in the children. All these conditions increase the language learners’ vulnerability in the dominant English-speaking world (Ishimaru, 2014).

While United States schools increase enrollment of multilingual learners, they simultaneously experience serious teacher shortages. Too many new teachers are unprepared for the classroom and especially lack experience working with diverse language learners and the trauma that can impact students from migrant and refugee backgrounds (Sutcher et al., 2016; McFarland et al., 2019). Among the certified teaching staff in the United States, only 2% of them are qualified to teach multilingual learners (U S Deoartment of Education, 2019). The median amount of training was only 4 h in 5 years for the certified teachers (Aud et al., 2011). Most language learners in the United States are struggling because they have little or no access to quality instruction tailored to their needs as indicated by the high number of untrained English language teachers (Lucas et al., 2008; Santos et al., 2012; Faltis and Valdés, 2016). In 2020, the population of United States students was trendy toward increased diversity with over half of the student population (54.34%) identified as having a diverse, cultural background (Irwin et al., 2022). These students, given their diverse and multilingual backgrounds, will greatly benefit from culturally and linguistically responsive instruction.

## 3 Social robots for language learners

When teachers have to attend to the needs of all the students, educational robots can play a critical role in engaging language learners in conversations. Modeling after human-human cross-cultural communications in classrooms, child-robot conversations need to demonstrate similar interactivity with culturally appropriate social cues that facilitate joint attention and rapport (Nomura et al., 2008; Trovato et al., 2013). These culturally appropriate social cues (Wiltermuth and Heath, 2009; Valdesolo and DeSteno, 2011) are crucial both to language learning (Meltzoff et al., 2009) and children’s willingness to engage with instructors (Harris, 2007; Corriveau et al., 2009). Prior work suggests that migrant children’s language development is about communicating meaning and having social interactions that enforces communication (Vygotsky and Cole, 1978; Richard-Amato, 1988; Irvine et al., 1992; Gass, 2017; Loewen and Sato, 2018). To encourage language development, we need to increase conversational language practice for migrant children at school (Kanda et al., 2004).

Social robots have been shown effective in supporting language development for children. Currently, social robots are used to provide tutoring (Leyzberg et al., 2014), increase language skills (Trovato et al., 2013; Westlund et al., 2015; Gordon et al., 2016), encourage storytelling (Mutlu et al., 2006; Fridin and Belokopytov, 2014; Westlund, 2015), and narrative interpretation (Han et al., 2009). Educational robots have been shown to increase children’s curiosity (Westlund, 2015), and encourage flexible thinking (Park et al., 2017). Language gains are found across multiple platforms, however children may prefer a social robot interaction over tablet or direct teacher interaction (Zhexenova et al., 2020). A few studies were conducted in Asia on English language acquisition showing promising results in elementary and middle school (Kanda et al., 2004; Nomura et al., 2008, 2016; Li et al., 2010). In the United States, social robots have shown to be successful in supporting language growth among preschoolers and elementary school students mainly in controlled lab environments (Breazeal et al., 2016; Kory Westlund et al., 2016; Belpaeme et al., 2018). Studies have also shown that social robots are more effective in language acquisition and retention than technologies such as a tablet, suggesting the potential of social robots in educational settings (Van Lier and Walqui, 2012; Vogt et al., 2017).

## 4 Cultural explorations of social robots

Numerous explorations have identified cultural differences in social robot preferences. Nomura et al. (Nomura et al., 2008) surveyed Japanese, Korean and American students and found that Japanese students held assumptions that humanoid robots had human characteristics and therefore their roles should be social, whereas Korean students had more negative attitudes of the social influences of robots. Similarly, Bartneck’s team (Bartneck et al., 2005) identified cross-cultural differences in attitudes towards robots by surveying Dutch, Chinese, German, Mexican, American (United States) and Japanese participants. They found that the Japanese were significantly more concerned about the negative effect of robots on society. Han et al. (2009) found different cultural receptivity towards tutoring robots among parents in Japan, Korea, and Spain. Korean parents were the least resistant to having robots as tutors in the classroom. Li et al. (2010) found that compared with German participants, Chinese and Korean participants were more engaged with the robot and perceived the sociable robots to be more likeable, trustworthy and satisfactory. In contrast, German participants preferred to use robots as tools or machines rather than as companions.

It is not surprising that cultural differences are found when exploring beliefs and preferences related to social robots. However, researchers have attempted to translate cultural differences into design recommendations leading to an extensive list of considerations. For example, Blanchard and Ogan (2010) emphasized the importance of considering students’ cultural backgrounds when designing a technology-based tutoring system. In order to enhance robots’ interaction with people of different cultural backgrounds, Trovato et al. (2012, 2013) recommended alignment between the nationality of the subjects and the cultural characterization of the robot’s facial expression, gestures, and ways of speaking such as greeting. Rau et al. (2020) called attention to cultural tendencies resulting in cognitive biases which designers must take into account in cross-cultural design to increase user comfort and accessibility. Nomura et al. (2016) suggested that designers thoroughly survey people’s expectations towards robots in the country where the robots are to be deployed. Although culture affects thinking and behaviors in daily life, very few insiders of a cultural community are conscious of how culture influences their being and their functioning. It is notoriously difficult to articulate cultural values that shape our likes and dislikes, what we feel comfortable with and what turns us away (Torreggiani, 2020). Designers, however, have to respond to users’ cultural values and knowledge to create educational robots that matter for the education of learners from diverse cultural backgrounds. For this reason, social robot behaviors and embodiments designed according to inquiries of cultural preferences, however, overlook the importance of *cultural responsiveness*.

## 5 Cultural responsiveness

Social robots may be an innovative tool to support diverse language learners, however, social robots must be designed to be culturally responsive. Culture consists of the meanings, behaviors, and practices that groups of people develop and share over time as well as the tangible expressions of a way of life, such as artifacts, values, and extent of explicitness in communication (Geertz, 1973). People from different cultural communities vary in the ways they express opinions and emotion (Hall, 1976), in their means of problem solving (Louie and Louie, 1999), and in their affordances of objects and design (Oshlyansky et al., 2004). For the past three decades, educators have embraced a culturally responsive pedagogy that enhances students’ academic growth and improve their psychosocial well-being (Paris and Alim, 2014; Gay, 2018; Keehne et al., 2018). Cultural responsiveness is observing and respecting the cultural characteristics, experiences, and perspectives of diverse individuals. Differences are considered as assets, which we encourage individuals to bring to their tasks or studies. This deserves distinction from *cultural appropriation* which is the unacknowledged or inappropriate adoption of the customs, practices, ideas of one people or society by members of another and typically more dominant society usually in a disrespectful manner. Although cultural responsiveness is slowly integrating into educational settings, integrating these characteristics into research is less common.

Sun (2012) proposes a new, innovative framework known as “culturally localized user experience” (CLUE) as a more robust framework to capture the dynamic nature of culture within technology design. This framework suggests a dialogic interaction between the designer and the users and views culture as a flexible and contextually-based entity. Cultural values are mutable and subject to the influences of the communities that surround those individuals. The difficulty of identifying an individual’s cultural profile is further complicated as the world (and schools) become more globalized. In addition, the increasing mobility of the contemporary world enables individuals to be exposed to and shaped by numerous cultural communities. Therefore, we can no longer depend on a narrow concept of preconceived and overly generalized characteristics to define and individual’s culture (Asher, 2008).

Sun (2012) articulates a practice-oriented vision of cultural differences, urging technology designers to invite diverse users to be co-designers to explore the features and interactions of technology that is intended to better serve them. The exploration stance encourages users to go through cycles of design-test-revise, giving them agency and permission to make changes on the robotic features and interaction design. Using a human-centered approach for interactive robot design takes into account the diverse users’ expectations, their capabilities, and their preferred behaviors. Therefore, building upon Sun’s theoretical framework, our series of three research studies depart from the traditional designer-determined approach to instead invite culturally and linguistically diverse learners and stakeholders as co-designers and expert informants in the design process. Each of the following examples illustrates and important key principle for designing with and for multicultural users.

## 6 Three research studies

The literature affirms the importance of recognizing cultural differences but design requirements for multicultural settings are limited and design principles are extremely rare for this population. Educational literature supports cultural responsiveness as a key component in working with multilingual learners. This leads to the question: *How can designers integrate cultural responsiveness in HRI and decrease the proximity between local users and the robotic design?* We suggest the CLUE framework (Sun, 2020), “working with local users in a local context” as an approach to explore the development of design requirements for multilingual learners. The missing element in previous efforts to understand and design for multicultural users is to bring the users as *expert informants* to the designing platform, engaging them as co-designers to close the gap between designers and the users. To gather and incorporate perceptions and preferences from a diverse setting, we use a this approach. Using a participatory design (PD) approach to explore the design requirements for social robots. This approach is appropriate and successful due to its commitments and meaningful engagement of people in the design process (Björling and Rose, 2019). In PD, the goal is not to just understand people in an effort to build systems for them, but rather to create co-operative and collaborative design relationships that can empower users and make practical or political improvements in people lives (Spinuzzi, 2005). Involving both culturally-diverse users (e.g., students) and stakeholders (e.g., parents and teachers) in the design process allows for improved and engaging design, improves the user experience, and results in greater academic gains and ownership of the technology-based learning.

In this next section, we introduce our three core principles for designing culturally responsive social robots. In addition, we illustrate the practice of each of these principles with a reference from our own work. We have successfully implemented each of these principles which helped us to understand how to design and implement educational social robots into classrooms with culturally and linguistically diverse language learners.

### 6.1 Principle 1: Gather Stakeholder Beliefs and Expectations

**Rationale:** This principle emerges from the vast literature on cultural responsiveness in education (Nieto et al., 1996; Paris, 2012; Ladson-Billings, 2014; Gay, 2018; Ishimaru, 2019). The culturally responsive literature suggests we must consider beliefs of family and community stakeholders who influence the student participant. Gathering data to illustrate the parents beliefs and expectations helps to articulate the cultural context for the learner. For multilingual learners, this also means that engaging and understanding the teachers who support the student is important to the process. Teacher beliefs and expectations directly shape the learning environment, and thus must also be heavily considered.

**Study 1:** Knowing that socio-cultural background influences the perceptions and values of a cultural group member (Vacca, 2019), we sought to address the following questions. 1) What robot images are preferred by language learners, parents, and educators? 2) How do teachers of language learners perceive the role and value of educational robots in their classrooms? 3) How do language learners, parents, and educators perceive the value of educational robots in a language learning environment? And 4) What concerns do the language learners, parents, and educators have regarding educational robots in the school setting?

**Method:** We explored the perceptions of robots among teachers, students and parents. Multilingual learners and their parents and teachers were recruited from an urban and diverse school district in the Pacific Northwest by the school-based liaisons of the professional training grant. These schools comprised 8–33% of multilingual learners and 60–90% of low income households. Interested parents were interviewed after an educational event at a local community center or before or after school based upon their preference. Parents were invited to group interviews by a school-based grant liaison during an educational session specific to these parents. Staff were invited by a school-based liaison to participate in individual interviews either before or after school based upon their choice. All adults consented prior to their interviews following IRB protocol. Given the language barriers for parents and our English-speaking research team, interpreters were used during the community center parent interviews. Individual and small group interviews took place in a classroom at their child’s school with a teacher present who also helped with language translation. Interviews ranged from 15 to 40 min depending upon the number of parents and their desire to share information. Parental consent and student assent were obtained before we interacted with the students at schools following IRB protocol.

**FIGURE 1:**
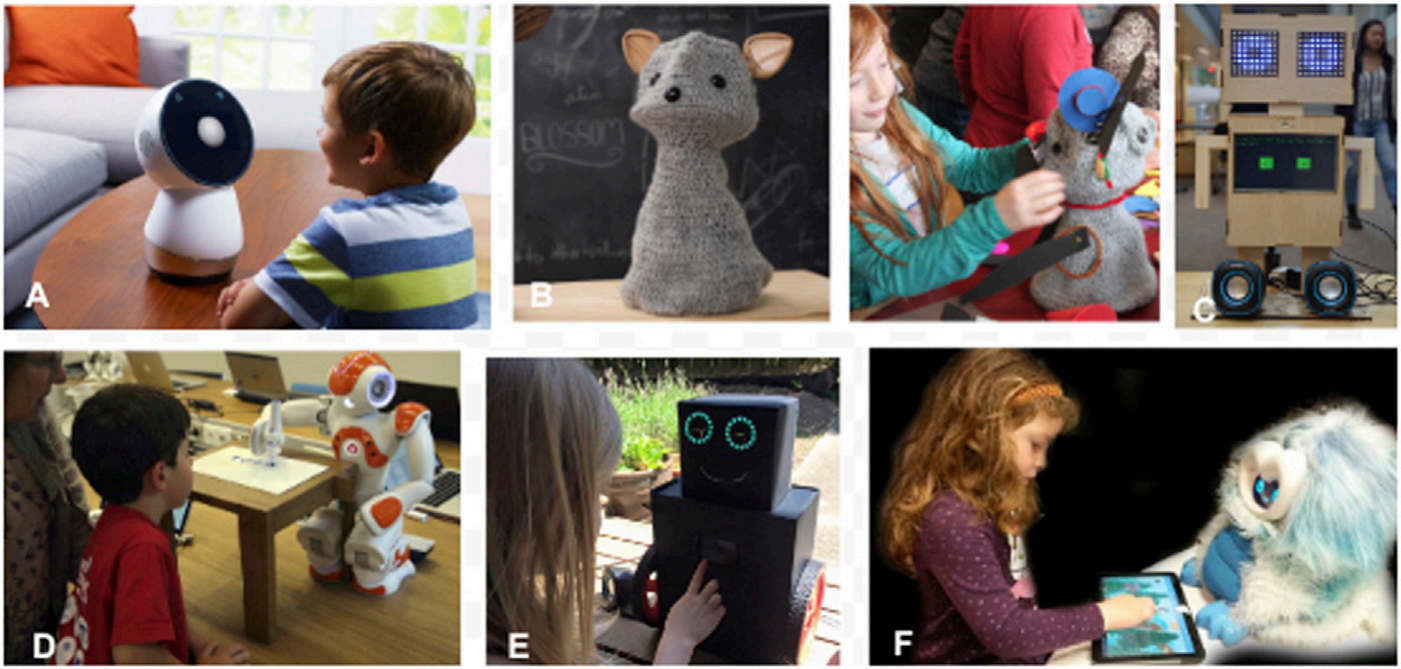
Participants responded to these educational robot images to determine their preferred robot. **(A)** Jibo, **(B)** Blossom, **(C)** EMAR V2, **(D)** Nao, **(E)** EMAR V1, and **(F)** Dragonbot.

We interviewed and surveyed 8 school educators as well as 95 multilingual learners from 17 language backgrounds and 39 language learner parents from 6 home language groups. Our goal was to understand what robot images were preferred by language learners, parents, and educators. We also wanted to know the participants’ perspectives on the role and value of educational robots in a language learning environment. Because robots were new to all the schools, it was important to identify the concerns that language learners, parents, and educators had regarding this technology. In one task, we shared images of 6 robots and asked the participants to select the robot that they felt was most ideal to have in their classrooms. The options were Dragonbot, Jibo, EMAR 1, EMAR 2, Blossom, and Nao. See Figure 1 for an illustration. For more details about this study, see (Louie et al., 2021).

**Results:** Nao was the top choice for many language learners and parents if they were to select an educational robot for their classrooms. Parents expressed their support, believing that shy students might be less embarrassed to practice speaking to a robot which was not judgmental. Teachers expressed support for what they saw as a powerful technology because robots would be less intimidating for children to interact with a robot than with an adult or their peers. Both parents and teachers were concerned about the possibility of robots replacing teachers in the classrooms. Some children were concerned that robots might misbehave by breaking things.

From this first study, we quickly learned that language learners had clear preferences about the appearance of a social robot. Many selected Nao because it looks like a real person. Both language learners and parents commented that Nao looked very smart, implying that Nao might be a good helper for the students. Some of them had a very negative reaction towards Dragonbot, saying that it looked scary. They rejected Dragonbot because of its appearance without finding out the functions of the robot or how it could support the students’ learning.

**The Principle:** Gathering stakeholder beliefs and expectations was essential to understanding the diversity of beliefs and expectations across home language groups and across stakeholder groups. For example, many Chinese parents suggested the importance of the social robot as an educational tool and that it not be perceived as a toy. This illustrated a different cultural value from some Spanish-speaking parents who suggested that the social robot might support their children’s social emotional needs as well as providing language practice. All of the parents were concerned about their language learners’ success in education in order to ensure economic success in the future. Without understanding the parents’ and teachers’ expectations of social robots, robotic designers will have problems delivering a product that satisfies the stakeholders. Learners will not be motivated to interact with an educational robot that their parents reject and their parents do not endorse.

### 6.2 Principle 2: Utilize non-verbal Co-Design methods

**Rationale:** The second principle is to **utilize to use non-verbal co-design methods** (e.g., drawing images, responding to photos, crafting embodiments). The National Academies of Sciences, Engineering and Medicine (National Academies of Sciences et al., 2018)recommend that multilingual learners be able to participate in STEM content areas Drawing from a desire to make our process inclusive and accessible, regardless of language proficiency, it was essential to ensure full participation from multi-lingual learners. Language proficiency should not be a prerequisite for learners to access technology (National Academies of Sciences et al., 2018).

**FIGURE 2:**
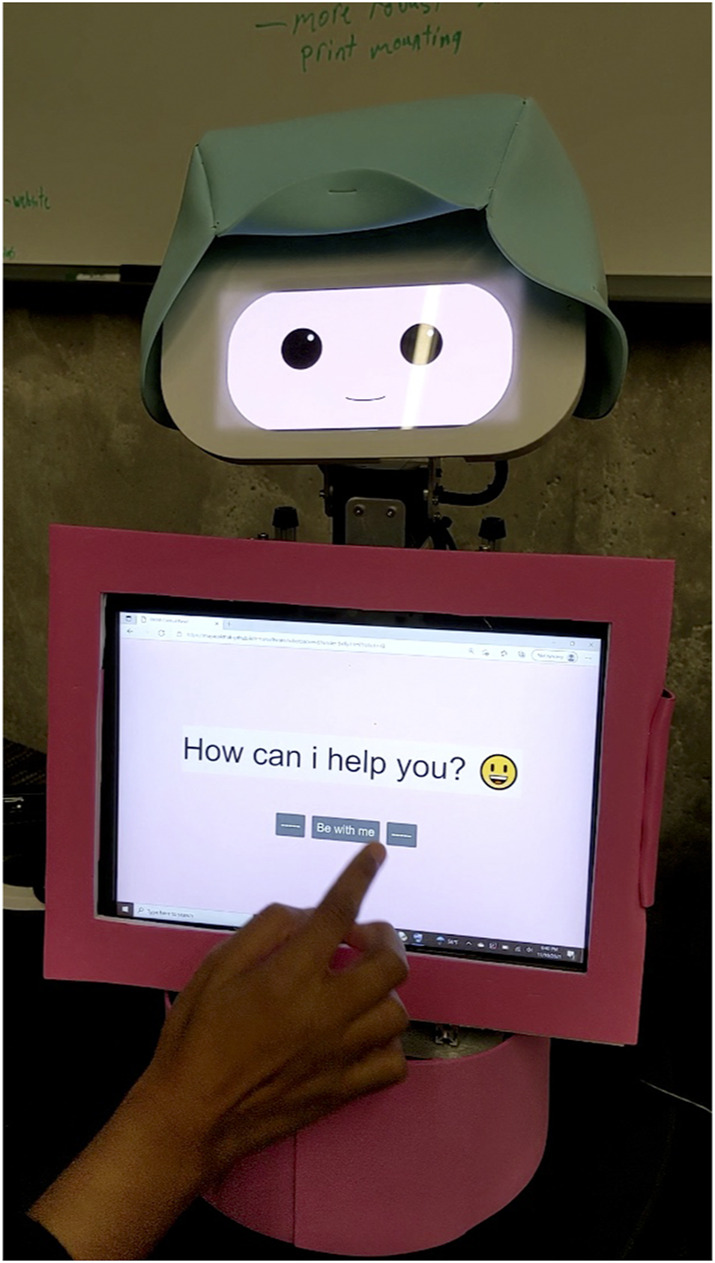
FLEXI is a mobile, flexible social robot system built for customization.

**Study 2:** Note: This study has not been previously shared or published. We wanted to explore co-design of a social robot intended for language practice in a summer camp environment with a diverse range of participants. We employed numerous non-verbal methods to ensure accessibility for multilingual learners of various English proficiency levels. We used FLEXI (Alves-Oliveira et al., 2022), a flexible and customizable robotic system which allowed co-designing and increased participation for language learners to integrate their cultural preferences in facial features, embodiment, and child-robot interaction. FLEXI is a social robot designed by Björling and Cakmak at the University of Washington. See Figure 2 As an interactive social robot, FLEXI has several advantages for language learners. This robot consists of a robust, table-top core with a customizable digital face, haptic sensors in its head, 4 degrees-of-freedom allowing for expressive movement (2 for the head, 1 for the neck, and 1 for rotating the whole body), and cameras which can be used as sensors or for video interactions.

Our research questions were:

• How would co-designing robotic features and interactions affect language learners’ engagement?
• How did language learners use language during their interaction with FLEXI in small groups?

**FIGURE 3:**
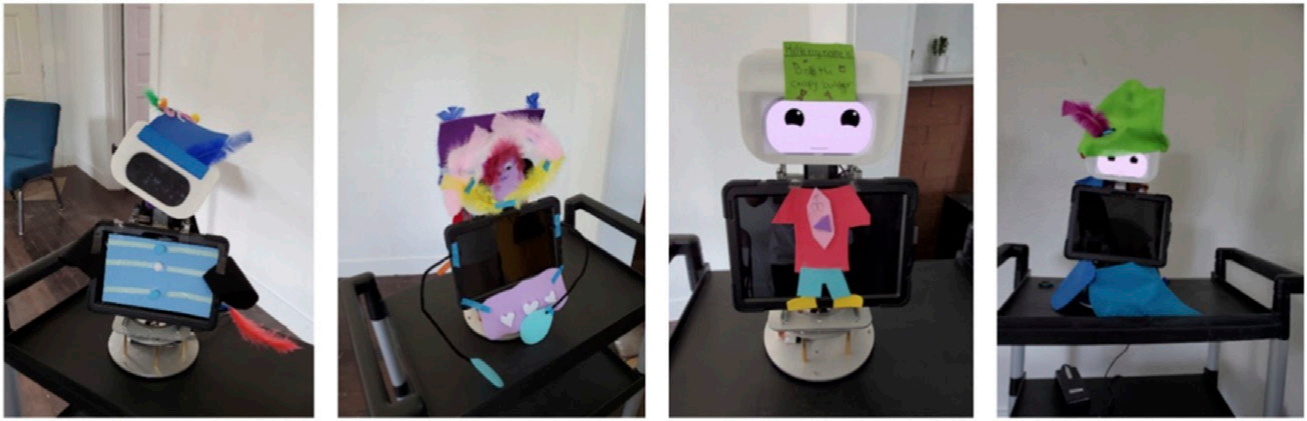
Four examples of language learner robot designs for FLEXI. Each design was very unique illustrating the diversity of ideas and desires of the language learner sample.

**Method:** We conducted four preliminary design sessions with the language learners (6–17 years old) at a summer camp to gather input about their impressions and ideas about customizing the robot’s features and embodiments. All the participants came from Chinese-speaking families and they varied in their English proficiency from limited to functional. Human subjects approval was obtained. The Summer camp helped us to inform parents and collect parental consent before the project started. Student assent was obtained in the summer camp with the help of an interpreter. We divided the language learners into four groups based on their age. The researcher asked the students to create a robot to use in the classroom to help students learn English. These design sessions included:

1) Face editing: The researcher introduced the FLEXI face-feature design program and demonstrated how students could choose from the collection design features to create the face of their robot. The students then worked in groups of 2 or 3 to do the design work for 20 min.
2) Embodiment design context: Students were told to design a body for this robot in 30 min using the craft materials and tools provided by the research team. language learners pretended that this robot was going to be in their school and help with language learning. Prompting questions were used: i. *What might its name be?* (Default “FLEXI” if students cannot decide), ii. *What should its color be?* iii. *What else would you like it to have?*, and iv. *What do you think about how it looks?*
3) Interaction design: Students were told to try interacting with the robot and see how it felt. The robot then asked students a few questions so they could see how it felt talking to the robot, for example FLEXI was programmed to say: *“Can one of you tell me a story about something that happened at camp last week …?”*
4) Debriefing: After students had the experience of interacting with their robot design, we asked them for informed feedback about the robot with question such as, What did you like about working with the robot? and What else would you want the robot to do?
5) Findings: i) Embodiment Design: In the co-designing sessions with FLEXI, all the students eagerly participated in group activities and discussed with their teammates. Throughout the whole session, students were very focused on the tasks at hand. The students’ embodiment designs were diverse and varied greatly. When students designed the embodiment for FLEXI, all the robot forms were human-like characters instead of animals or abstract. See Figure 3. The language learners liked their FLEXI robot designs. They also expressed that FLEXI appeared to be responsive to their stories due to its blinking eyes and tilting its “screen” head. They used the term “cute” to describe the facial features and embodiment they selected and created.
ii) Language use: Not surprisingly, children of various language proficiencies were equally engaged with the facial feature customization process. Because all of them spoke Chinese, the limited language proficiency level students discussed with their teammates in Chinese or a mixture of Chinese and English to discuss what to select and how to create. The hands-on designing activities provided an accessible context for the language learners to communicate. The translanguaging practice demonstrated that the language learners are using their available language capacity in multiple languages to focus on the task at hand. “Languaging” is important because we tend to talk when we problem-solve as languaging can be a means to focus our minds. Robotic design, even though it was new to all of the language learners, did not trigger fear and hesitation. Instead, the group design process became a catalyst for language use.

Some students asked whether they could change the kinds of verbal remarks FLEXI might give them in addition to saying *“Thank you!”* Words of encouragement and compliments are another form of cultural responsiveness to multilingual learners (Fong, 1998; Winner, 1999; Mkhitaryan and Babayan, 2020).

### 6.3 Principle 3: Provide an experiential robot interaction

**Rationale:** The third principle is **provide an experiential component** of child-robot interaction to create a more accessible method by which to elicit feedback and ideas while minimizing the language demand. The hands-on exploration allows for previously unvoiced or unknown preferences to emerge. In addition, it provides ecological validity by allowing child participants to speak directly from experience of a child-robot interaction, rather than speaking theoretically. These experiences also allow child participants to express feelings resulting from interactions such as comfort levels.

**Study 3:** Given the importance of cultural responsiveness in human–human interactions in the classroom, our third study was an exploration of translating a culturally responsive interaction onto a social robot designed to work with language learners. Importantly, we were also interested in providing an actual social robot interaction for multilingual learners to gain a more contextual response. For more detailed information about this study, see (Björling et al., 2021). Having learned from a previous study (Louie et al., 2021) that language learners, their teachers and families preferred the Nao robot form factor, we chose to use the Nao robot for this study. It was also imperative to conduct the study in a real-world setting to better understand how interactions might differ from expectations. We investigated how language learners perceived and interacted with a Nao robot conducting a culturally responsive discussion in their classrooms. In an effort to leverage these language learners as cultural informants in the process, we hoped to answer the following questions: 1) How do language learners experience interactions with a culturally responsive, social robot in a small group discussion setting? 2) After experiencing the social robot interactions, what modifications or iterations do language learners suggest to improve their experience and make the social robot more culturally and contextually appropriate? And 3) How do experienced second language teachers evaluate culturally responsive robot interactions with language learners?

**Method:** After obtaining institutional human subjects review approval, we recruited a second language teacher as a collaborator to work with us. Child-robot interactions took place during the school day based upon the classroom teacher’s preference. Parents were informed of the study taking place in their child’s classroom during the school day and consented to study and the use of their child’s image for research publications. We used a participatory design approach to conduct an exploratory study with 24 Spanish-speaking third grade to fifth grade language learners in an elementary school. Fourteen third-graders and ten fifth graders, (ages ranging from 8 to 10, 12 boys, and 12 girls) participated in this study. All students spoke Spanish as their home language and were receiving special pull-out English language instruction with English proficiency learners ranging from level 1 to 3 according to the state WELPA test (McGraw-Hill Education, 2020). It is important to note that although all of the multilingual learners had the same home language, they were still quite diverse in relation to their home cultures stemming from numerous South American countries. The researcher met with them in groups of 3 for 15-min in their regular classroom for their pull-out English language instruction. The students were reminded that their participation was voluntary and they could withdraw anytime without any penalty. As cultural informants, students participated in a 15-min, robot-led, small group story discussion to give them a robot interaction to elicit their ideas and feedback about the robot. Each student appeared engaged and completed the interaction and the interview that followed. During the meeting, students responded to Nao’s questions on a story read to them by the researcher before the meeting. All the sessions were videotaped, transcribed, and analyzed.

Second language teachers were recruited through a Pacific Northwest urban school district *via* word-of-mouth. Six school staff (teachers, instructional coaches, a bilingual liaison, and an administrator) from local area elementary schools participated in the reflexive critique interviews. Many of the instructors had up to 30 years of teaching experience spanning different age groups from children to adults and working in environments across the United States and abroad. All the teachers were currently teaching or directing English language programs in a Pacific Northwest, urban public school district.

**FIGURE 4:**
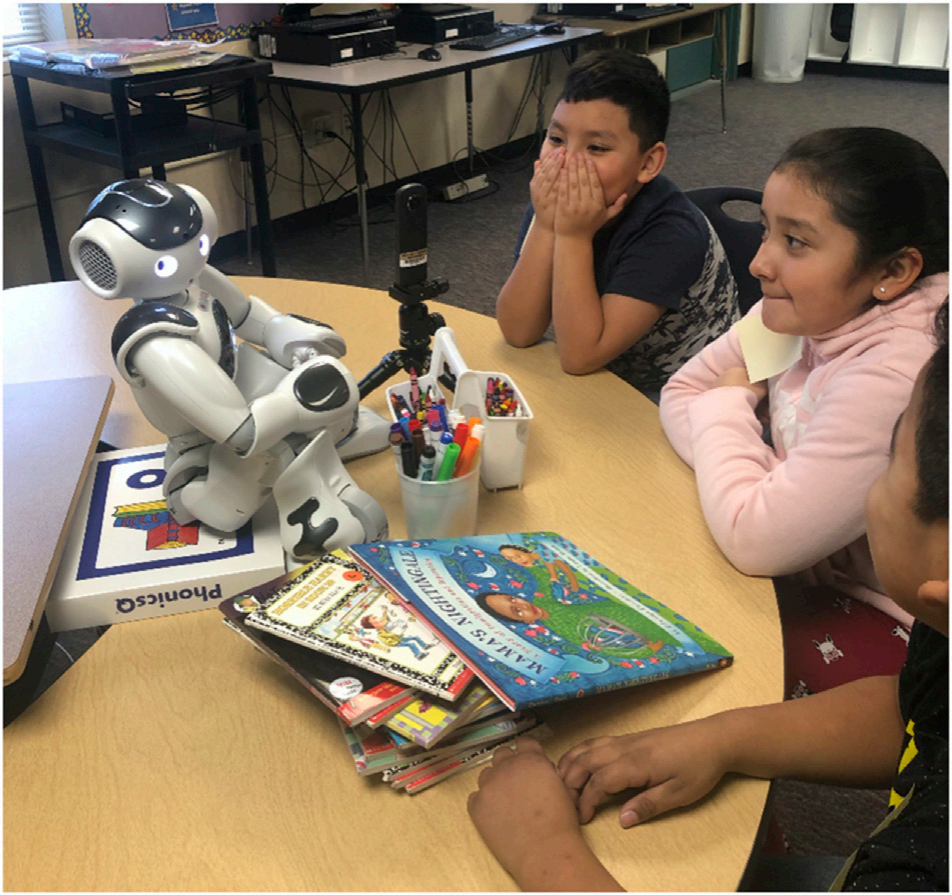
Three English Language learners are interacting with the Nao robot.

**Results:** During the story-telling activity, students were fairly quiet, sometimes covering their mouths with shyness or surprise (Figure 4). However, during the discussion phase of the activity, many students became talkative and were excited to share their ideas. In addition, we saw students translate and help one another in sharing their responses to the robot.

The elicitation of feedback from the students (to discuss what could be altered to make them feel more comfortable) as cultural informants was a culturally responsive activity and resulted in strong engagement. In fact one student who was silent during the interaction, was incredibly talkative when asked to voice her preferences. Although her English was limited, she engaged another student to translate her feelings for her into English such that the researchers would understand her experience. Utilizing a participatory approach while engaging learners in the actual experience of interacting with a social robot provided them some context from which to respond about how they felt and what they desired. Involving language learners in design decisions for social robots is a promising way to engage language learners in a meaningful interaction with the robots. The design conversations stimulated meaningful and relevant language use among the learners.

**The Principle:** Our Nao interaction study helped us to see that students were more engaged and responsive in the debriefing sessions than they were in the language interactions. When they were given the power to suggest changes, the invitation encouraged them to open up. Language learners seldom had the opportunities to give personal input regarding devices which did things to them, for them, or with them. The rich conversations suggest that co-design activity itself may motivate students to communicate and provide enough value to facilitate discussion among language learners, a sorely needed yet infrequent occurrence with the language learners in classrooms.

## 7 Discussion of guiding principles

Current robotic design for diverse users is designer-determined, presuming that diverse learners can adapt to the universal robotic design or there are general and stable cultural traits for people populations as published in the literature (Hall, 1976). Universal, one size fits all design principle rarely works for any consumers’ products and could be excluding for diverse users such as multilingual learners. There is no surprise that a robot designed to function in a certain pattern will not fare well with a diverse population of users. Given the global reach of technology, the cultural beliefs and outlooks of diverse users have been influenced by what they are in contact with. Cultural traits and beliefs are dynamic instead of stable, changing with the experience of the users. Furthermore, diverse users from same country origins now migrate to different parts of the world through diverse pathways. These once-upon-a-time kinfolks are now myriad of cultural beliefs and behaviors being changed by the journey. We can no longer attach the same cultural traits to people with the same country origin.

In this paper, we proposed three guiding principles to support culturally responsive social robot design. We illustrated how these principles informed our own studies with culturally and linguistically diverse language learners in educational settings. These principles are grounded in a combination of cultural responsiveness, participatory design, and the CLUE Framework, all of which encouraging designers to center their users and to consider and design for the explicit contexts in which the users reside. All three principles are essential to ensure that social robot design is both appropriate, accessible and meets the needs of the many multilingual learners in our educational settings. The National Academies of Sciences, Engineering and Medicine (National Academies of Sciences et al., 2018) voices serious concerns about equitable access to STEM technologies and robotics for multilingual learners. However, in addition to equitable access, multilingual learners also need culturally responsive curriculum and devices - including social robots.

*Principle 1: Gather Stakeholder Beliefs and Expectations*, We studied the perceptions and expectations of the three major groups of stakeholders for multilingual learner education. Educators understand the significance of baseline assessment before any instruction. Understanding multilingual learners’ perceptions and expectations of educational robots are part of this baseline assessment. Such knowledge must be gathered before any curriculum creation, technology design, and instruction delivery to ensure a good fit of the educational efforts. As we are going to place educational robots in classrooms where teachers preside over all the decision making, it is only reasonable to understand teachers’ expectations. Educational robots will only be successful if their support aligns with the goals and expectations of the teachers. Multilingual learners’ homes are acknowledged as viable resources to be leveraged in the classroom. Parents can be active stakeholders in their children’s education (Upadhyay, 2009; Buxton, 2010; Fournier, 2014; Zeichner et al., 2017). Research suggests that building strong connections between teachers and families and communities creates mutual understanding, which can improve multilingual learner opportunities and motivation to engage in STEM learning (Moll et al., 1992; Baquedano-López et al., 2013; Ishiguro and Dalla Libera, 2018).

*Principle 2:Utilize Non-Verbal Co-Design Methods* We utilize non-verbal co-design methods to engage students and to ensure that English proficiency is not a prerequisite to robotic education. Not only did this make our project accessible to learners regardless of language ability, but it also seemed to encourage the desire for verbalization (in both English and home languages) and peer-supported translation from multilingual learners given during periods of debriefing. As cultural informants, learners make suggestions for iterations on the social robot interaction. The students’ input directly informed new interaction behaviors and verbalizations for the Flexi system.

*Principle 3: Provide and Experiential Robot Interaction* We provided an experiential robotic interaction to multilingual learners as they need a strong and immediate context in order to provide embodied, experiential feedback regarding their preferences and their experience. In our first study, we gathered feedback about robots in general from numerous stakeholders. However, human-robot interaction is a novel, and embodied experience that is difficult to imagine. For this reason, learners need to experience the robot interaction. Just the physical presence of a social robot has been shown important for learning gains (Leyzberg et al., 2012). In addition, interactions with the social robot in their real-world setting provides rich environmental context, which is important in social robot design (Šabanović et al., 2014).

We feel strongly that these principles not only ensure that cultural context is elicited during data collection, but also that the design process is culturally responsive. A participatory approach to social robot design may be imperative to designing appropriate and inclusive social robot embodiments and features for diverse participants such as language learners. But regarding child participants as cultural informants may be equally important. The act of participating in co-design activities and making space for diverse learners to voice their preferences allows for agency and engagement that may be far less common in other their classrooms. As we have illustrated in our own interdisciplinary work with language learners, a participatory, co-design approach has proved successful in both leveraging the voices and engagement from our participants, but also in allowing us to gather valuable data about the preferences of our population. As we move toward educational robots as a complementary tool to provide students with increased support and engagement, we must encourage designers, educators, and parents to do so utilizing a participatory approach. In order to enhance teachers’ willingness to use robots to support diverse learners, we need to honor the cultural and linguistic assets learners bring to the classrooms by integrating input from all three essential stakeholders of the educational context, the students, their parents, and the teachers.

## Data Availability

Anonymized data supporting the conclusion of this article will be made available upon request.
